# Post-Mortem Examinations

**Published:** 1910-05-07

**Authors:** 


### The Hospital

A Journal of

The Medical Sciences and Hospital Administration.

New Series. No. 168, Vol. VII. [No. 1240, Vol. XLVIII.] SATURDAY, MAY 7, 1910.

### POST-MORTEM EXAMINATIONS.

There are few duties so unpleasant to the medical practitioner as that of urging upon the unwilling and distressed relatives of a deceased person the importance of performing an autopsy to verify diagnosis or to explain some unforeseen and ill-interpreted complication. Not only is the task of overcoming such a reluctance among the friends a difficult and ungrateful one, but in private general practice, particularly in the country, it involves also, as a rule, the personal conduct of the post-mortem examination, a business which is lengthy, unremunerative, and not even always useful if he who carries it out has allowed his pathological knowledge to get rusty. Yet a third disadvantage is that unless great tact and care be exercised, both in obtaining the required consent and in conducting the investigation with due reverence, there may arise a tendency for the sick to cease to consult the particular man whose conscientiousness impels him to ask for an autopsy in doubtful or obscure cases; and this may operate in a most unfavourable way, especially if others of the neighbouring medical men are sufficiently opportunist to try to make capital out of the sentimentality of the public in this matter. Yet, all these disadvantages notwithstanding, there are those who do make successful efforts to obtain the requisite sanction fairly frequently; and Professor Sir Clifford Allbutt, in one of his writings, mentions the case of a country practitioner who rarely failed to do a complete examination of every patient who died while under his care.

In hospitals the difficulties are not so great. Facilities for pathological work include up-to-date mortuary and post-mortem room accommodation, at least in our larger city hospitals, though the Reports upon Hospitals which have appeared in these pages during the past year demonstrate very eloquently the pressing need for improved conditions in these respects in the smaller provincial hospitals. Expert pathologists are entrusted with the conduct of autopsies, and there is no fear of the hospital officer incurring odium or loss of revenue through his making the necessary overtures to the relatives. Yet even in hospitals, as all those who have filled the post of resident medical officer are well aware, the most violent and invincible opposition is not at all uncommon. Here again the value of tact, sympathy, and persistence is inestimable; there are some resident medical officers who very rarely fail to obtain permission for examination, and there are others who succeed only when dealing with the most complaisant or enlightened. Moreover, opposition from the friends of a patient drawn from the hospital class is the outcome of prejudice, due much more often to ignorance than to sentimentality, and an explanation of the proceedings—that is, the progress of scientific medicine with the view of rendering curable or remediable that which at present is neither—throws an entirely new light upon the case and is nearly always sufficient to procure the desired consent. It has always been said that at one large and well-known hospital every patient on admission is provided with a copy of the rules, one of which is to the effect that in the event of death the hospital authorities shall have leave to submit the body to autopsy if they think fit. Whether that is actually done or not, the idea is one which has distinctly something to recommend it.

To the profession it is a commonplace that the medical student and the recently qualified hospital resident learn more from the autopsy-room than from any other one single department of the hospital. But to the public it is naturally not so obvious that this is the case, though the great interest with which the progress of cancer research and other eagerly expected advances are watched for has probably diffused among the laity a wider appreciation of the methods of scientific medicine than formerly existed. When it is fully and popularly comprehended that there is nothing mysterious about the making of a diagnosis, that brilliant intuitions are not the stock-in-trade of the safe physician, but that careful and patient investigation of every factor in the case, full knowledge of the import of each, and a careful judgment every step in the formation of which should be capable of reasoned explanation; when all this penetrates the average individual's head, then it will be more fully realised how enormously the interpretation of observed symptoms is assisted by an actual inspection of the diseased parts in which they have been displayed. It is probably not an overstatement to say that the consulting physician attached to the staff of a hospital surpasses the general practitioner in the analysis and synthesis of physical signs to an extent strictly proportional to the extra facilities for post-mortem study which he enjoys necessarily and of which he has availed himself. A careful and thorough autopsy conducted by an expert gives beyond question the best possible explanation of the clinical phenomena observed during life, reveals the limitations as well as the strong points of the methods of observation employed, and serves to facilitate correct diagnoses in similar conditions should the attendant physician come across them in his future practice. Since all treatment worthy of the name is founded upon correct diagnosis, it can scarcely be denied that the practice of carrying on research by the help of post-mortem examination is one which stands second, in the benefits conferred upon mankind, only to animal experimentation as a means of therapeutic advancement.

With that sane and practical common-sense for which we nowadays look mainly to Germany, though it was formerly thought to be almost a monopoly of the English, the Germans have recognised the importance of autopsies from an educational point of view. There is no doubt that in general German physicians enjoy greater facilities for the study of morbid anatomy than does the profession in England, and this is doubtless partly the result of their own efforts. Too often the British doctor allows some deceased patient to be buried on a certificate which bears at best a haphazard guess at the exact lesion which has determined death. Sometimes because he shirks the preliminary bother with the relatives, sometimes because he does not want to take the trouble of making the examination, and sometimes because he is afraid that people in his district will fight shy of him to his own detriment and the advantage of his professional neighbours. The solution of the whole matter is, of course, education: the education of the public to the necessity of autopsies for the increased usefulness of the profession, and of the practitioner to the realisation of the fact that he is and must ever remain a learner all his life.

